# Re-induction of hormone sensitivity to diethylstilboestrol in androgen refractory prostate cancer patients following chemotherapy

**DOI:** 10.1038/sj.bjc.6604052

**Published:** 2008-01-08

**Authors:** R A Cox, S Sundar

**Affiliations:** 1 Department of Clinical Oncology, Nottingham City Campus, University of Nottingham Hospitals NHS Trust, Nottingham NG5 1PB, UK

**Sir**,

The majority of patients with hormone sensitive prostate cancer become hormone refractory over time. Recently, Shamash *et al* (2005) reported on the reinduction of hormone-sensitivity in androgen refractory prostate cancer patients following chemotherapy with lomustine and chlorambucil. In their prospective study, eight out of 17 (47%) patients who were rechallenged with hormonal therapy following the failure of chemotherapy had a prostate specific antigen (PSA) response. Interestingly, 50% (four out of eight) of the patients responded to rechallenge with diethylstillboestrol (DES). We have had a similar experience in two patients following chemotherapy with docetaxel and prednisolone.

## PATIENT A

**Figure 1:**
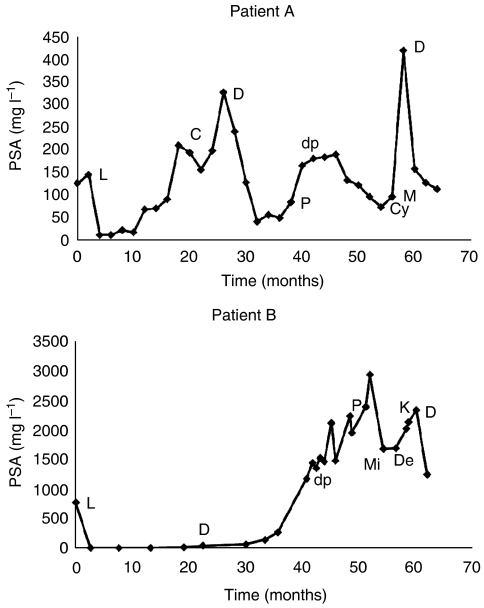
The PSA response to treatment in patients A and B. C (cyproterone acetate 100 mg tds); Cy (cyclophosphamide 50 mg daily); D (diethylstilboestrol 1 mg daily); DP (docetaxel and prednisolone 10 mg daily); K (ketoconazole and hydrocortisone); L (LHRH agonist – Goserelin 10.8 mg); Me (megace); Mi (mitozantrone started); P (prednisolone 10 mg daily).

A 66-year-old man presented with acute urinary retention in July 2002 with a PSA of 125 *μ*g l^−1^ and was diagnosed with a Gleason 8 adenocarcinoma of the prostate. The PSA fell to 9.9 *μ*g l^−1^ after commencing indefinite Goserelin 10.8 mg implants; cyproterone acetate (CPA 100 mg tds) was started 21 months later on PSA relapse (see Figure 1). In December 2004, the patient was admitted with painful swelling of his left leg with an ultrasound Doppler excluding a deep-vein thrombosis but a CT scan showed significant left pelvic lymphadenopathy. The PSA had once again relapsed so the CPA was changed to DES (1 mg daily) with aspirin (75 mg daily) – the PSA initially decreased then remained stable for 12 months. In January 2006, prednisolone (10 mg daily) was unsuccessfully tried following further PSA increase and so the patient was given chemotherapy with docetaxel and prednisolone. On completion of six cycles in August 2006, the prednisolone was continued with the addition of cyclophosphamide (50 mg daily). Megace was unsuccessfully trialled in December 2006, following further relapse. In February 2007, the patient was rechallenged with DES leading to a marked and sustained response with the PSA falling from 420 *μ*g l^−1^ to the most recent value of 112 *μ*g l^−1^ six months later.

## PATIENT B

A 72-year-old man presented in February 2002 with a PSA of 824 *μ*g l^−1^ and was diagnosed with a Gleason score 10, bone-scan-positive adenocarcinoma of the prostate. The PSA fell to a nadir of 0.5 *μ*g l^−1^ after starting indefinite goserelin implants, with cyproterone acetate (50 mg daily) later prescribed for hot flushes. In Spring 2003, the patient had a subtotal colectomy for T3N0M0 colon carcinoma that did not require any adjuvant treatment. In 2004, DES was started following hormone relapse, but the PSA continued to progress. After palliative radiotherapy to the spine, the patient received docetaxel and prednisolone chemotherapy with a good clinical and biochemical response. Six cycles of chemotherapy were completed by January 2006 and the patient maintained on prednisolone (10 mg daily). A single high PSA in February 2006 was thought to be secondary to acute urinary retention and subsequent catheter insertion. Following further PSA relapse, the patient completed six cycles of mitozantrone chemotherapy, and then subsequently failed to tolerate ketoconazole and hydrocortisone. Most recently, the PSA has fallen from 2333 to 1244 *μ*g l^−1^ following rechallenge with DES.

## DISCUSSION

A criticism of the study by Shamash *et al* (2005), is that the patients were not androgen suppressed during chemotherapy, and therefore responses to the reinduction of hormone therapy may have been due to the growth of androgen-dependent clones. However, two patients with a previous orchidectomy, and therefore castrate during chemotherapy, responded to rechallenge with DES in their study. Our two patients described above, continued androgen suppressive therapy throughout their treatment and this suggests that DES may also have an androgen-independent mechanism of action (Pienta and Bradley, 2006).

Chemotherapy with docetaxel and prednisolone has now become the standard treatment for hormone refractory prostate cancer (Tannock *et al*, 2004). There are currently no standard treatment options for patients after the failure of docetaxel (Berthold *et al*, 2005). Based on our experience and that of Shamasa *et al*, we suggest that rechallenge with DES is a worthwhile option for androgen-refractory prostate cancer patients relapsing after chemotherapy.
